# Proteome dynamics and early salt stress response of the photosynthetic organism *Chlamydomonas reinhardtii*

**DOI:** 10.1186/1471-2164-13-215

**Published:** 2012-05-31

**Authors:** Guido Mastrobuoni, Susann Irgang, Matthias Pietzke, Heike E Aßmus, Markus Wenzel, Waltraud X Schulze, Stefan Kempa

**Affiliations:** 1 Max Delbrück Center for Molecular Medicine Berlin, Berlin Institute for Medical Systems Biology (BIMSB), Berlin, Germany; 2 Max-Planck-Institute for molecular plant physiology Potsdam-Golm, Berlin, Germany

## Abstract

**Background:**

The cellular proteome and metabolome are underlying dynamic regulation allowing rapid adaptation to changes in the environment. System-wide analysis of these dynamics will provide novel insights into mechanisms of stress adaptation for higher photosynthetic organisms. We applied pulsed-SILAC labeling to a photosynthetic organism for the first time and we established a method to study proteome dynamics in the green alga *Chlamydomonas reinhardtii*, an emerging model system for plant biology. In addition, we combined the analysis of protein synthesis with metabolic profiling to study the dynamic changes of metabolism and proteome turnover under salt stress conditions.

**Results:**

To study *de novo* protein synthesis an arginine auxotroph *Chlamydomonas* strain was cultivated in presence of stable isotope-labeled arginine for 24 hours. From the time course experiment in 3 salt concentrations we could identify more than 2500 proteins and their H/L ratio in at least one experimental condition; for 998 protiens at least 3 ratio counts were detected in the 24 h time point (0 mM NaCl). After fractionation we could identify 3115 proteins and for 1765 of them we determined their *de novo* synthesis rate. Consistently with previous findings we showed that RuBisCO is among the most prominent proteins in the cell; and similar abundance and turnover for the small and large RuBisCO subunit could be calculated. The D1 protein was identified among proteins with a high synthesis rates. A global median half-life of 45 h was calculated for *Chlamydomonas* proteins under the chosen conditions.

**Conclusion:**

To investigate the temporal co-regulation of the proteome and metabolome, we applied salt stress to *Chlamydomonas* and studied the time dependent regulation of protein expression and changes in the metabolome. The main metabolic response to salt stress was observed within the amino acid metabolism. In particular, proline was up-regulated manifold and according to that an increased carbon flow within the proline biosynthetic pathway could be measured. In parallel the analysis of abundance and *de novo* synthesis of the corresponding enzymes revealed that metabolic rearrangements precede adjustments of protein abundance.

## Background

*Chlamydomonas reinhardtii* belongs to the green algae and is the most widely used laboratory strain of the *Chlamydomonas* genus. Besides being a model organism for the study of fundamental biological questions, this species also gains more and more interest as a model for systems analyses and biotechnological applications. The release of the *Chlamydomonas reinhardtii* genome sequence in 2007 [1] set the basis for systems analyses and genome-wide studies and introduced *Chlamydomonas* as model organism; since then several systems level analyses have been performed. By applying metabolomic and proteomic analyses the genome annotation of *Chlamydomonas* was refined [2]. Based on the results from metabolomic studies, missing reactions from the metabolic network could be inferred [3] and ChlamyCyc, a web portal for systems analyses, was generated [4]. Also a stoichiometric genome-wide metabolic network from *Chlamydomonas* was constructed, that enables flux balance analyses [5]. However, such networks rely on stoichiometric basis and do not contain regulatory properties of the pathways. Furthermore, not all regulatory mechanisms are discovered to date.

Understanding the dynamics and stability of biological systems requires deeper insights in the temporal regulation of cellular processes. Specifically, the response of the photosynthetic apparatus to changing environmental conditions can be studied on a cellular level. Salt stress is detrimental to plant growth and increasing salt contaminated areas cause problems in agriculture. Thus, understanding adaptation strategies of higher plants to salt stress is of major importance. Furthermore, a better understanding of the salt stress response of green algae may allow engineering strains with an improved resistance to high salinity. Such strains could be cultivated in salt containing water but would keep the desired properties.

To date, methods are available and have been constantly improved to analyze the dynamics of the proteome, the transcriptome and the metabolome. Techniques for monitoring the dynamic changes within the proteome and metabolome are mostly based on metabolic labeling of metabolites and proteins using stable isotopes such as ^13^ C or ^15^ N in combination with mass spectrometry or nuclear magnetic resonance (NMR) analyses. Most techniques were initially developed to analyze microorganisms or mammalian cell cultures, e.g. the mass isotopomer ratio analysis of u-^13^ C labeled extracts (MIRACLE) or the stable-isotope labeling in cell culture (SILAC) using arginine and lysine [6,7].

Arginine and lysine are essential amino acids for many higher organisms and are commonly used for SILAC-based proteomic studies. However, plants have the capability to synthesize all proteinogenic amino acids. Thus the application of stable-isotope labeled arginine in plants results in a partially labeled proteome [8,9]. To completely label plant proteomes ^15^ N sources were applied [10].

In mammalian cells stable isotope labeled amino acids were applied to determine *de novo* protein synthesis [11,12]. However the application of ^15^ N sources for pulse labeling resulted in an enormous complexity of the isotopic pattern of partially labeled proteins and peptides [13]. By improving the data analysis workflow Martin and co-workers could analyze the dynamic ^15^ N incorporation into 92 peptides resulting in 40 proteins [14]. Other metabolic labeling techniques have also been used, however, only a limited set of plant proteins were monitored [15]. Recently, stable isotope labeled arginine has been used for metabolic labeling of proteins in *Chlamydomonas* to study proteome-wide response to anaerobic growth conditions [16].

In the present study we introduce a strategy to measure protein synthesis rates in *Chlamydomonas*. For the first time pulsed labeling with ^13^ C-labeled arginine was applied in a photosynthetic organism. We thereby utilized the ability of *Chlamydomonas* to take up arginine as carbon and nitrogen source from the environment [17].

With this method we characterized the salt stress response of *Chlamydomonas*. The response of the micro alga *Dunaliella salina* to high salt stress was already analyzed on proteomic level [18]. Also the salt adaptation of *Arabidopsis thaliana* on metabolome level was investigated in a time course manner [19,20]. In contrast, the salt stress response of *Chlamydomonas* was not studied systematically.

Applying pulsed SILAC and GC-MS based stable isotope resolved metabolomics [21] we identified the major metabolic pathways altered in the early response of *Chlamydomonas* to salt stress. We observed that the proline-biosynthetic pathway displayed a strong response at the metabolite but not at the protein level. Our study is the first application of a combined proteomics and metabolite approach to study the stress respons of a green alga and represents the first comprehensive analysis of the early salt stress response of *Chlamydomonas reinhardtii*.

## Results

In this study we analyzed the protein synthesis rate in *Chlamydomonas reinhardtii* by pulsed labeling using stable ^13^ C6 arginine. For this purpose we made use of the arginine-auxotrophic *Chlamydomonas* strain CC-1618, containing the mutated arg2 allele of the ARG7 gene. ARG7 encodes for the argininosuccinate lyase that catalyzes the last step of arginine biosynthesis [22,23]; consequently this strain requires arginine in the culture medium.

As a first step, we compared the incorporation of ^13^ C arginine into the proteome of a wild type *Chlamydomonas* strain (CC-125) versus the arginine auxotroph strain (CC-1618). We found an incorporation of ^13^ C arginine in both strains but the rate of incorporation was higher in the arginine auxotrophic strain (Additional file 5: Figure S1). Hence, for all further experiments the arginine auxotrophic strain was used.

**Figure 1:**
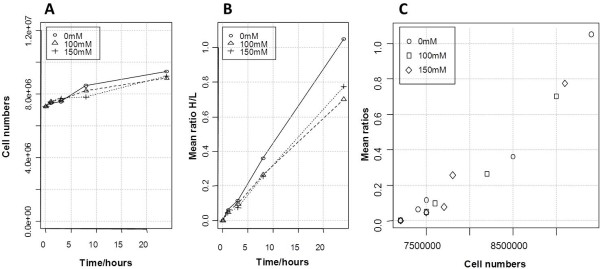
**Cell growth and incorporation of stable isotope labeled arginine.** Relation between observed protein ratio and cell number at 0 h, 1 h, 3 h, 8 h and 24 h after addition of stable isotope labeled arginine upon 0 mM, 100 mM and 150 mM NaCl; **[A]** cell numbers,** [B]** mean of all protein ratios and **[C]** mean ratios plotted against cell numbers at all conditions.

*Chlamydomonas* cells were cultivated in three different conditions (0 mM, 100 mM and 150 mM NaCl) and harvested in a time course manner 1, 3, 8 and 24 hours after the supply of ^13^ C arginine. For this experiment, *Chlamydomonas *cells were cultivated in the condition of constant and slow growth (see Materials and Methods and Figure 1A). Under the chosen conditions the cell number increased by a factor of 1.3 after 24 hours. We have chosen these conditions to better distinguish *de novo* protein synthesis rates – related to the increase in biomass – from real protein turnover. Upon salt stress cell growth was mildly reduced (Figure 1A). In total, samples from 12 conditions were directly analyzed in duplicates and the protein heavy/light ratio (H/L ratio) for several hundreds of proteins in any of the samples could be calculated by the MaxQuant software [24]; a constant increase of the mean protein ratio was observed for all conditions (Figure 1B); also growth behavior and mean H/L ratio displayed a linear co-behavior (Figure 1C). The H/L ratio reflects the protein synthesis rate since only newly synthesized proteins can incorporate ^13^ C-labeled arginine. 998 proteins with three or more reported H/L ratios (ratio count) could be obtained from the 24 h 0 mM time point; to classify the proteins into functional classes the MapMan annotation was applied [4,25] and this protein set was used for further analyses (see Additional file 1: Table 1 for the complete list of proteins and the supplemental material).

**Figure 2:**
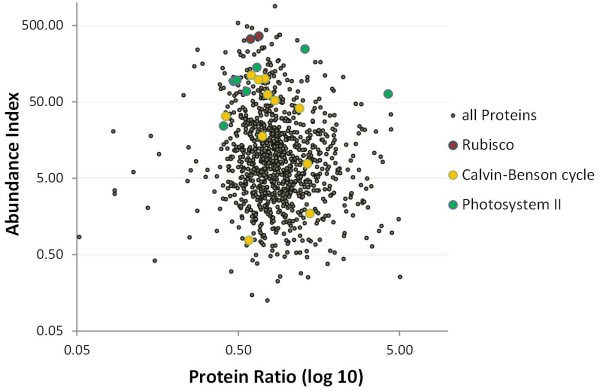
**Genome scale overview of protein abundance and relative protein synthesis rates.** Plot of protein abundance index versus protein H/L ratios from a proteomic analysis of *Chlamydomonas reinhardtii* (strain CC-1618) grown under standard conditions and metabolic labeling of proteins with ^13^ C-labeled arginine for 24 hours. All proteins with more than 4 ratio counts are shown in the diagram; Histones, RuBisCO and Photosystem II proteins (PS II) were selected and highlighted.

We used this dataset to rank relative protein abundance (Abundance Index, AI) by dividing the measured intensity by the molecular mass of the protein. This simple calculation is comparable to the emPAI index [26], with the difference that the latter index is calculated as (10^(PAI) -1), where PAI is the ratio observed/observable peptides for each identified protein. This calculation allows an intra-sample protein abundance comparison. To test how reproducible this calculation may work we analyzed a mixture of 48 recombinant proteins distributed over an abundance range of 6 orders of magnitude. As it is reported in Additional file 2: Table 2 and Additional file 5: Figure S 2, the abundance values calculated for proteins at each concentration level show a very narrow distribution. We extracted the quantitative values of photosystem II (PSII) proteins and of the small and large subunits of RuBisCO from the control time course data set. In agreement with literature data, which report RuBisCO subunits being the highest expressed enzymes of the Calvin-Benson cycle, the AI values for both subunits are among the top 10 in the dataset; moreover, these values reflect a nearly perfect 1:1 stoichiometry between the small nuclear-encoded and the large chloroplast-encoded subunits (Figure 2). Similarly, also proteins of the photosynthetic apparatus appear among the most abundant ones. Furthermore, we extracted the AI values and the H/L ratios for the proteins of the photosynthetic apparatus in the 0 mM NaCl condition from the time course dataset, in order to monitor their behavior.

**Figure 3:**
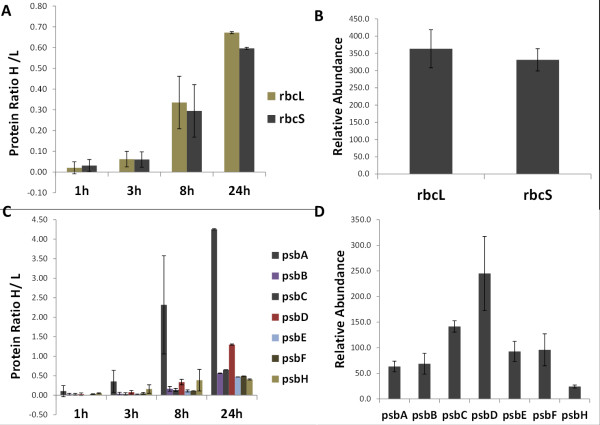
**Time dependent incorporation of stable isotope labeled arginine and protein concentration.** Reported are the H/L ratios and relative protein abundance of RuBisCO large (rbcL) and small subunit (rbcS) as well as of photosystem II proteins. [**A**] Protein ratios of rbcL and rbcS at 0, 1, 3, 8 and 24 h from control conditions (0 mM NaCl) are shown with the standard deviations of all observed H/L ratios of all corresponding peptides. [**B**] Bars show the average abundance of rbcL and rbcS from the 0, 1, 3, 8 and 24 h time points (0 mM NaCl). [**C**] Protein ratios of photosystem II proteins at 0, 1, 3, 8 and 24 h from the control condition (0 mM NaCl). [**D**] Bars show the average relative abundance of photosystem II proteins from the 0, 1, 3, 8 and 24 h time points (0 mM NaCl).

**Figure 4:**
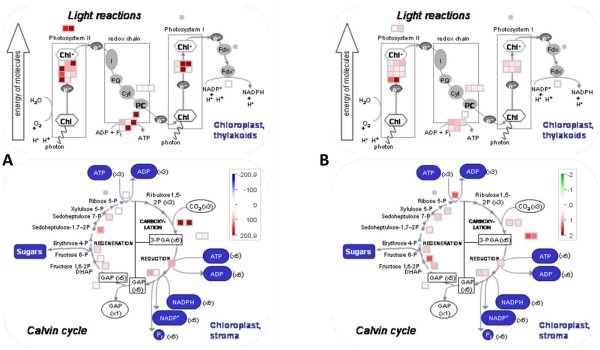
** Abundance and turnover of PSII and Calvin-Benson-cycle proteins.** MapMan visualization of the proteins of the functional categories ‘light reactions’ and ‘Calvin-Benson-Cycle’ with their [**A**] respective H/L ratios and [**B**] relative abundance.

The H/L ratios for RuBisCO large (rbcL) and small (rbcS) subunits were found constantly increasing in a similar way, indicating comparable synthesis rate and stability (Figure 3A), whereas the relative abundance was constant and with low standard deviation over the entire time course (Figure 3B). Among the proteins with high synthesis rate we found psbA, also termed ad D1 protein, which showed a synthesis rate higher than the other PSII proteins across the entire time course (Figure 3C and 3D), in agreement with Pick and co-workers [15]. A list of selected proteins with the corresponding H/L ratios for the 24 h time point and the AI is reported in Table 1; PSII proteins and proteins of the Calvin-Benson cycle are shown in Figure 4. The complete list of the proteins identified is available in the supplementary information (Additional file 1: Table 1).

**Table 1:** Relative synthesis rates of selected proteins

	Identifier	MapMan Bincode	Description	Protein Ratio	SD	Ratio count	Abundance index	SD
Protein with high Ratios	143757		N/A	5.0	+/- 0.5	3	0.3	+/- 0.5
	186587		N/A	4.9	+/- 0.5	4	1.5	+/- 0.5
	195524	19.1	magnesium chelatase	4.5	+/- 3.8	5	1.0	+/- 0.3
	191409		N/A	4.4	+/- 3.1	5	21.8	+/- 28.0
	194316	18	co-factor and vitamine metabolism	4.4	+/- 3.2	15	34.2	+/- 17.2
	psba		psba	4.3	+/- 1.9	12	63.3	+/- 10.2
	195711	9.2.2	mitochondrial electron trtansport/ATP synthesis	3.9	+/- 3.3	13	8.2	+/- 5.1
	189624	1.1.40	cyclic electron flow- chlororespiration	3.9	+/- 0.1	3	1.2	+/- 0.7
	134058	34.16	ABC transporter	3.8	+/- 1.2	16	9.6	+/- 3.6
	177538		N/A	3.7	+/- 4.6	12	1.0	+/- 0.6
	129468	12.2.2	glutamine synthase	3.6	+/- 3.5	7	20.5	+/- 13.6
RUBISCO	rbcL	1.3.1	RuBisCO large subunit, rbcL	0.7	+/- 0.1	32	363.1	+/- 55.1
	108283	1.3.2	RuBisCO small subunit, rbcL	0.6	+/- 0.1	15	331.4	+/- 32.3
	193086	1.3.13	rbcL N- methyltransferase	1.4	+/- 0.4	4	1.7	+/- 1.1
	128745	1.3.13	RubisCO activase	1.2	+/- 0.2	12	41.0	+/- 11.8
Calvin Benson Cycle	135614	1.3.11	Ribulose phosphate-3-epimerase	0.7	+/- 0.2	5	17.7	+/- 6.7
	195910	1.3.12	Phosphoribulokinase	1.3	+/- 0.4	6	7.7	+/- 4.0
	24084	1.3.7	Fructose-1,6-bisphosphate	1.4	+/- 0.0	2	2.3	+/- 1.6
	24459	1.3.6	Fructose-1,6-bisphosphate aldolase	0.8	+/- 0.3	18	51.9	+/- 16.7
	141319	1.3.8	Transketolase	0.8	+/- 0.2	23	62.1	+/- 14.8
	129019	1.3.4	Glyceraldehyde 3-phosphate dehydrogenase A	0.7	+/- 0.1	20	100.7	+/- 13.7
	132210	1.3.3	Phosphoglycerate kinase	0.7	+/- 0.5	16	96.8	+/- 8.2
	102889	1.3.4	Glyceraldehyde 3-phosphate dehydrogenase, nonphosphorylating	0.6	+/- 0.1	5	0.8	+/- 0.6
	189186	1.3.9	Sedoheptulose-1,7-bisphosphatase	0.6	+/- 0.2	31	111.3	+/- 21.9
	26265	1.3.5	Triose phosphate isomerase	0.4	+/- 0.1	7	32.4	+/- 8.2
Photosystem II	psbA	1.1.1.2	psbA	4.3	+/- 1.9	12	63.3	+/- 10.2
	psbB	1.1.1.2	psbB	0.6	+/- 0.2	25	68.6	+/- 20.3
	psbC	1.1.1.2	psbC	0.7	+/- 0.1	29	141.3	+/- 11.1
	psbD	1.1.1.2	psbD	1.3	+/- 1.2	15	244.8	+/- 72.3
	psbE	1.1.1.2	psbE	0.5	+/- 0.1	3	92.7	+/- 19.7
	psbF	1.1.1.2	psbF	0.5	+/- 0.1	4	95.8	+/- 31.2
	psbH	1.1.1.2	psbH	0.4	+/- 0.1	4	24.2	+/- 2.9

Protein list with the corresponding H/L ratio at 24 h (0 mM NaCl) and abundance index (AI) across the entire time course. Proteins are sorted in four functional groups: high synthesis rates, RuBisCO, Calvin-Benson cycle and Photosystem II; Joint Genome Institute (JGI) protein identifier, MapMan bincode, a protein description, protein ratio and relative abundance index are reported for each protein.

**Table 2:** Salt included changes of protein synthesis

Protein IDs		Annotation	MapmanBin	Normalised protein ratio		
				0 mM	100 mM	150 mM
up regulated protein ratios	147180	NA	NA	-3.83	-2.62	-2.15
	134305	redox ascorbate and glutathione	21.2.1	-4.54	-2.95	-2.93
	131444	S-assimilation, APR	14.2	-2.52	-1.08	-1.05
	196360	serine protease	29.5.5	-1.09	0.16	0.16
	149072	NA		-0.76	1.03	0.49
	119219	PGM	4.2	-0.61	0.61	0.58
	130434	valine degradation	13.2.4.3	-0.64	0.21	0.48
	196738	FAP24		1.40	2.57	2.48
	129593	Methionine degradation	13.2.3.4	-0.28	0.75	0.77
	185032	NA		-0.11	0.98	0.92
	162449	Phosphoglycerate dehydrogenase	13.1.5.1.1	0.04	0.96	0.93
	24552	Carbonic anhydrase	8.3;8.3	-1.63	-0.76	-0.74
	123507	serine protease	29.5.5	-1.25	-0.18	-0.37
	187392	Branched chain amino acid synthesis	13.1.4.1	0.13	1.31	1.00
	191617	Asparate-tTNA ligase	29.1.12	-0.11	0.74	0.73
	137300	Starch phosphoylase	2.2.2.2	0.06	1.15	0.88
	123463	Auxin metabolism	17.2.1	-1.31	-0.41	-0.54
	195255	Carbamoyl-phosphate synthase	13.1.2.3.11	-1.30	-0.33	-0.54
down regulated protein ratios	126820	Malate dehydrogenase	8.2.10	0.45	-0.70	-0.64
	16132	FMG1-2		-2.45	-3.60	-3.54
	33411	Oxygen-evolving enhancer protein2	1.1.1.2	0.32	-0.91	-0.81
	24392	Protein postranslational modification	29.4	0.65	-0.54	-0.59
	1670	Protein targeting secretory pathway	29.3.4.99	1.68	0.56	0.42
	148898	HPC2		-0.77	-2.16	-2.03
	205988	HSP70g	20.2.1	1.47	-0.07	0.18
	205649	PSII polypeptide	1.1.1.2	0.05	-1.21	-1.32
	187761	Protein degradation	29.5.9	1.23	-0.05	-0.22
	132633	Protein folding	29.6	1.40	-0.18	-0.20
	190311	FHA transcription factor	27.3.48	-0.38	-2.07	-2.05
	112806	Protein assembly and cofactor ligation	29.8	1.75	-0.47	-0.19
	196855	Stress related	20.2.1	1.63	-0.24	-0.31
	205900	PSII polypeptide	1.1.1.2	0.90	-1.16	-1.25
	183363	LHC-I	1.1.2.1	-1.35	-3.79	-3.85

Proteins with increased or reduced protein ratios at 24 h at the 5% level from 100 mM and 150 mM NaCl compared to proteins from the control culture (0 mM). The measured protein ratios were transformed (z-transformation) to enable a comparison of protein ratios from all conditions.

To monitor a larger set of peptides - and subsequently proteins - we used a novel method for isoelectric-fractionationof peptides in solution [27]. In contrast to other isoelectric-focusing techniques, this method allows fractionation of any protein or peptide sample in the desired pH range within less than 3 hours with high sample recovery. With this method we analyzed the samples taken at 8 and 24 hours in absence of NaCl in the culture medium. The fractionation prior the downstream MS analysis allowed us to identify more than 23.000 different peptides (versus 12,500 identified from all the 12 samples without fractionation) with a false discovery rate (FDR) of 1%. The identified peptides could be mapped to 3115 proteins identified with at least 2 unique peptides and for 1765 of those a H/L ratio was measured with at least 3 ratio counts in both conditions (Additional file 3: Table 3).

### Calculation of protein half lives

To analyze protein dynamics for a large number of proteins we measured several time points, from 1 to 24 hours after addition of ^13^ C arginine. Hypothesizing that within 24 h no protein underwent degradation and was just produced according to the increase of biomass, all proteins would display a protein H/L ratio of 0.3 (expected protein ratio). In contrast, the average ratio from all proteins was calculated to be 1.0, with values up to 6.0, thus a reasonable *de novo* protein synthesis in *Chlamydomonas* under this condition could be evidenced. Only a few proteins were found with a ratio below 0.3. Those proteins may not be synthesized at the same speed as biomass is increasing (Figure 2). Assuming exponential growth, an exponential decay of the light protein present at 0 h time point and 100% ^13^ C arginine incorporation into newly synthesized proteins, the half-life of proteins can be estimated [28]. Taken the increase in biomass of 0.3 times within 24 h and the assumption of exponential growth (P(t) = P0 * 2 t/Tdouble) resulted in a doubling time (Tdouble) of 63 h. For the robust calculation of protein half-lives we used the individual H/L ratios at 3 h, 8 h and 24ht. We applied simplified linear regression (includes the time point 0 h with a H/L ratio of zero) and calculated the half-lives of 710 proteins (supplementary table 4).

### Chlamydomonas Protein synthesis rates and metabolic alterations upon salt stress

**Figure 5:**
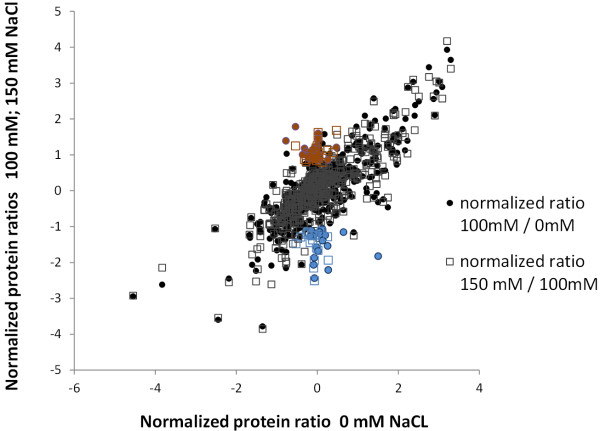
**Relative protein synthesis rates upon salt stress.** Normalized protein ratios obtained from *Chlamydomonas* after 24 h incubation with stable isotope labeled arginine upon salt stress (100 mM and 150 mM NaCl). Normalized protein ratios of 24 h/100 mM and 24 h 150 mM are plotted against protein ratios obtained after 24 h/0 mM NaCl. The upper and lower 5% of proteins with altered protein ratios at both salt conditions are marked with colors (red - up regulated and blue – down regulated).

**Figure 6:**
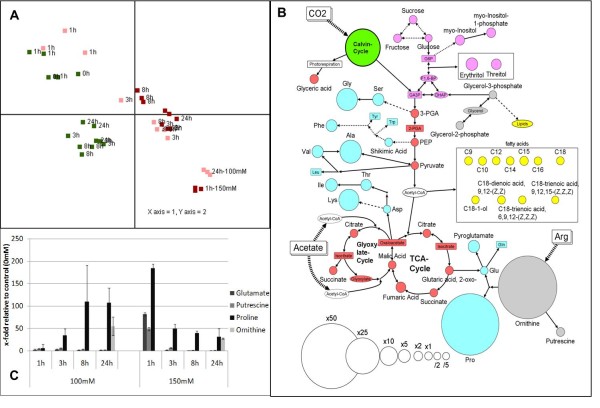
**Pathway from arginine to proline and stable isotope incorporation in metabolites.** Scheme of the metabolic pathway from arginine to putrescine, glutamate and proline; shown are the metabolites and reactions indicated by the gene names including their corresponding JGI identifier. Putrescine, glutamate and proline are highlighted with yellow background color; for those metabolites the ^13^ C stable isotope incorporation was measured by GC-MS the results are shown below the pathway scheme for each experimental time point and sodium chloride concentration. All proteins shown in black including the ARG7 protein could be identified by LC-MS/MS based proteomics at the 24 h/0 mM condition.

To analyze the salt stress response we cultivated *Chlamydomonas* with ^13^ C-arginine in standard growth medium containing 0 mM, 100 mM or 150 mM sodium chloride. The samples were collected in a time course manner after 1 h, 3 h, 8 h, and 24 h. Each sample was analyzed by LC-MS based proteomics and GC-MS based metabolomics. From the proteomics analysis we could determine the relative protein synthesis rates of several hundred proteins (Additional file 1: Table 1). For all conditions we observed an H/L ratio increasing over time, with a slope depending on the salt concentration (Figure 1B). The average synthesis rate was highest under the control condition (0 mM) and the lowest at 150 mM sodium chloride. The protein ratio was clearly correlated to the increase of cell numbers at each time point (Figure 1C). To compare synthesis rates from 0 mM, 100 mM and 150 mM the H/L ratios from the 24 h time point from each condition were Z-transformed and plotted against each other (Figure 5). This transformation corrects for the underestimation of the H/L ratio because of the metabolic labeling of proline and glutamate (Figure 6). The overlay of normalized protein ratios from 24 h/100 mM and 24 h/150 mM NaCl compared to control conditions revealed a similar behavior of proteins upon the two stress conditions (Figure 5); even the ranking of proteins with up or down regulated H/L ratios is the same (Table 2).

### Effects of salt stress on metabolism

**Figure 7:**
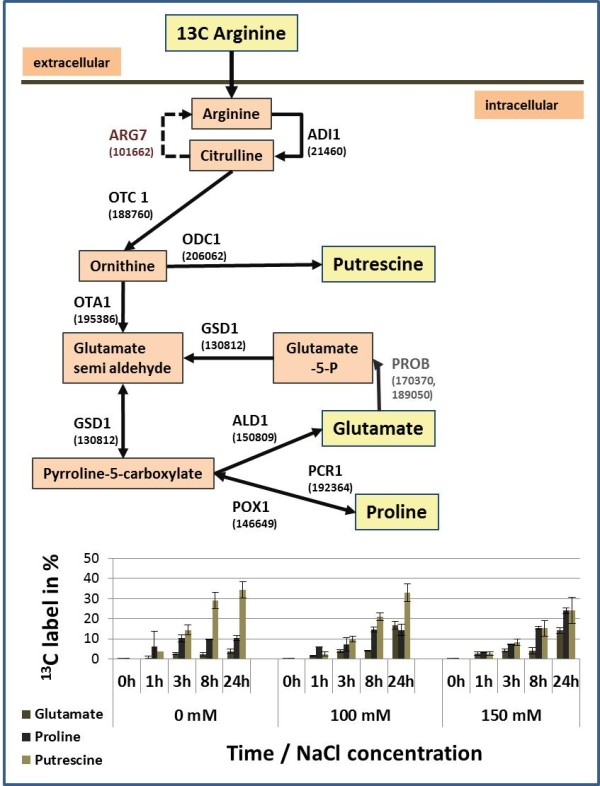
**Dynamics of metabolite adjustments to salt stress.** Overview about the metabolic changes of *Chlamydomonas* upon salt treatment; [**A**] principal component analysis (PCA) visualizing the metabolic changes measured by GC/MS based metabolic profiling, [**B**] relative changes of metabolite levels in *Chlamydomonas* after 24 hours treatment with 100 mM NaCl compared to 24 h time point of the control. The colors indicate different classes of metabolites: red and pink, intermediates of glycolysis and TCA cycle; light blue; amino acids, gray; others. [**C**] The temporal behavior of the amino acids proline, putrescine, glutamate and ornithine is shown at all time points compared to control (0 h/0 mM).

To monitor the metabolic response to salt stress a GC-MS based metabolome analysis was performed. With this technique we could identify the salt stress response at the metabolite level. To visualize the general behavior a principal component analysis (PCA) was performed; the PCA clearly separated samples treated with 100 mM or 150 mM NaCl from the control samples. Interestingly, the 24 h/100 mM sample showed a very similar metabolite pattern compared to the sample taken after 1 h at 150 mM (Figure 7A). Many metabolites showed a comparable response to salt stress. For example, at 100 mM NaCl proline concentrations reached a maximum after 24 hours, however, the same levels were reached at 150 mM NaCl already after 1 h (Figure 7A and 7C). These results indicate that *Chlamydomonas* can slowly adapt to 100 mM NaCl, but displays a fast shock reaction at 150 mM NaCl concentration, concerted by a strong metabolic response. The overview graphic (Figure 7B) displaying the relative metabolite changes after 24 h/100 mM compared to the 24 h non-stressed control shows that a strong response can be observed in the amino acid metabolism. We have extracted the isotopomer distribution from the GC-MS mass spectra, as described in [21], and have calculated the changes of ^13^ C abundance for several metabolites. Careful inspection revealed that ^13^ C carbon atoms derived from ^13^ C arginine could be detected in downstream amino acids (Figure 6).

Already after 3 hours the percentage of ^13^ C label in proline was 10% and in glutamate 3% and then stayed constant up to 24 h under control conditions (0 mM). Upon salt stress the ^13^ C-label was enhanced in proline and glutamate but not in putrescine compared to the control condition. Furthermore, upon salt stress proline levels increased substantially but glutamate levels only moderately indicating a distinct regulation of proline synthesis. Interestingly, the detected enzymes from the arginine to proline inter-conversion pathway did not show changes in abundance (Additional file 5: Figure 3).

## Discussion

Using pulse labeling of proteins with stable isotope labeled arginine we were able to analyze the half-life of more than 710 proteins, and the relative synthesis rate of 1765 proteins in *Chlamydomonas reinhardtii*. This is the first report of a systems-wide analysis of protein dynamics from a photosynthetic organism. The new established method allows monitoring dynamics of protein synthesis and thus will open new perspectives for a better understanding of biological processes. In addition technical procedures have been improved enabling a higher sample throughput. Our improved method for in-solution peptide fractionation [27] prior to analysis on a nanoLC-LTQ-Orbitrap MS system yielded a high number of identified peptides similar to other proteomics analysis of *Chlamydomonas*[16], but in a much shorter time period. Thus, our approach is suitable for comprehensive proteome analyses in a higher throughput manner. The data were used to calculate the relative abundance of proteins allowing for an inter-protein abundance comparison. In our study both RuBisCO subunits rbcL and rbcS showed a nearly perfect stoichiometry under the chosen conditions (Figure 2B). Also the half-life for both subunits was found comparable (Figure 2A). This is remarkable because rbcL is transcribed and translated in the chloroplast, while rbcS is encoded in the nucleus. This suggests the existence of regulatory mechanisms aimed to ensure a similar synthesis rate, highlighting the co-regulation of nuclear/cytosolic and chloroplastic processes. The currently available methods for absolute quantification of proteins as absolute SILAC [29] or quantitative targeted proteomics methods [30] are selective for defined proteins and thus are not applicable for discovery experiments. We observed constant protein concentrations at all time points under control conditions and thus assumed a steady state of our system (Figure 3B3D). Ongoing from this assumption we calculated a median protein half-life of 45 hours. Interestingly, this result is comparable to the median protein half life of mammalian cells of 46 h [28], which display a much higher proliferation rate. The protein synthesis rates in *Arabidopsis thaliana* were analyzed using ribosome occupancy analyses and quantitative proteomics [31]. Even if the approaches are not directly comparable the estimated protein synthesis rates are in the same dimension. We could identify proteins with high *de novo* synthesis rates. Interestingly, a high turnover rate of the psbA protein was already observed more than two decades ago [15] and nowadays the regulatory nature of this protein in response to high light stress is well understood [32].

The intensity of the ^13^ C labeled peptides may be underestimated because of the interconversion of ^13^ C arginine into ^13^ C proline and its subsequent incorporation into newly synthesized proteins. In our study we could observe only a minor fraction of peptides containing ^13^ C proline; furthermore, the maximal ^13^ C label of the free proline pool was 10% and the fraction of ^13^ C labeled glutamate was below 3% upon control conditions. Such a high degree of ^13^ C proline incorporation as observed by Heide and co-workers [33] could not be evidenced in our study. Thus we assume that the presented method is suitable to determine protein dynamics in experiments up to 24 h.

We investigated the dynamics of the salt stress response of *Chlamydomonas* by an untargeted metabolome analysis. In particular we compared the protein turnover and the metabolic rates of the proline biosynthetic pathway. The metabolome analysis revealed that amino acid metabolism is strongly induced in *Chlamydomonas* upon salt stress. At a concentration of 100 mM NaCl a stepwise adaptation from 1 h to 24 h took place but already at 1 h/150 mM a salt shock reaction was observed. At 150 mM NaCl metabolite levels showed an immediate response at the 1 hour time point. This metabolic reaction clearly preceded proteome rearrangements and thus it may be driven by yet undiscovered posttranslational regulation. With increasing salt concentrations the label incorporation into proline and glutamate increased whereas the label in putrescine was comparable at all 3 conditions. This observation may be explained by the pathway structure (Figure 6) and the regulation of the flux trough this pathway. Hence, these data clearly suggest posttranslational activation of enzymes of proline biosynthesis and thus, rerouting of metabolism at high salt stress in *Chlamydomonas reinhardtii*.

## Conclusion

Using large scale biochemical analyses it is possible to decipher regulatory steps within the cellular metabolic network, specifically the regulated enzymes and their mode of regulation. These studies will also help understanding stress induced metabolic alterations in higher photosynthetic organisms. In *Chlamydomonas* we found a proline accumulation upon salt stress; this reaction was also reported in higher plants and other algae [20,34,35]. Increases in proline and in mannitol could be detected after short term salt stress in the brown alga *Ectocarpus siliculosus*[36] and in *Nicotiana tabacum* prolonged salinity induced progressive accumulation of proline and myo-inositol [37]. It was shown that artificially increased proline levels in *Arabidopsis thaliana* plants resulted in an improved resistance against freezing and high salinity [38] for review see: [39]. This indicates that the substantial proline accumulation might be a functional response in *Chlamydomonas* to counteract salt stress. Interestingly, carbohydrates were not found up-regulated in *Chlamydomonas*. We observed that the metabolic patterns of the two different salt concentrations were not reflected in the behavior of the proteome and that the proteomic responses to 100 mM or 150 mM were very comparable (Figure 5). Thus, we may provide evidence that metabolic rearrangements can happen within a short time period if necessary, whereas proteomic changes are more conservative. The observed phenomena may be an expression of a basic regulatory concept in biology; to provide metabolic flexibility with the expressed set of proteins and to restrict ‘expensive’ rearrangements of the proteome to long term environmental changes. Similar phenomena were reported within the yeast metabolism [40]. These results highlight the power of posttranslational regulation in order to adjust metabolic processes to the demand of the cell. The new established method enables the determination of protein turnover in *Chlamydomonas* and will allow gaining deeper insights in dynamic regulatory processes of photosynthetic organisms; the presented strategies and data will be beneficial for basic research, biotechnology and for agricultural improvements.

## Methods

### Culture conditions and sampling

The *Chlamydomonas reinhardtii* strain CC1618 (cw15, arg7, mt-) was stored on Tris–Acetate–Phosphate-medium (TAP) agar plates, supplied with 10 mM arginine at 22 °C and a continuous light intensity of 150 μE/m2. Pre-cultures were grown in liquid TAP medium with 20 mM Tris-buffer and 10 mM L-Arginine up to OD of 1 on a rotary shaker at 110 rpm and 100 μE/m2s continuous light intensity. The pre-culture was pelleted at 600 g for 2 min and dissolved in Hepes-Acetate-Phosphate (HAP) medium containing 5 mM HEPES as a buffer and 10 mM ^13^ C6-labelled Arginine (Fluka). The starting OD of the experiment was 0.7; for the salt stress analysis two other cultures were grown in the same medium with addition of NaCl to a final concentration of 100 mM and 150 mM. For all measurements samples were taken from two independently grown cultures at the same time points (of 1 h, 3 h, 8 h and 24 h) after NaCl supply

### Proteomic sample preparation

2x50 ml of the cell-culture were collected and centrifuged for 10 min at 4 °C and the pellet was frozen. Cell pellets were resuspended in 600 μl of buffer Urea 8 M (Roth), ammonium bicarbonate 100 mM (Fluka). Cells were homogenized in Dounce homogenizer; samples were then shortly centrifuged and the supernatant filtered using Whatman filters (0.45 um pore size). Protein concentration was determined by Bradford assay (Pierce); an average yield of 1 mg of protein per sample was obtained.

The disulfide bridges of each protein sample were reduced in DTT 2 mM (Sigma) for 30 minutes at 25 °C and successively alkylated in iodoacetamide 11 mM (Sigma) for 20 minutes at room temperature in the dark. The samples were then diluted with 1.5 volumes of ammonium bicarbonate 100 mM and incubated with LysC endoproteinase (WAKO) for 7 hours at 37 °C; after LysC digestion the samples have been further diluted with 1.5 volumes of ammonium bicarbonate 50 mM and incubated with 10 μl of immobilized trypsin (Applied Biosystems) under rotation for 16 hours at 37 °C. After digestion all the samples have been desalted on Stage Tips [41] prior to LC-MS analysis. Samples from all experimental conditions were analyzed in duplicates.

For the isoelectric fractionation of peptides in solution 600 μg of protein digest were desalted on SPE cartridges (3 M) following manufacturer instructions. The eluted peptides were then dried under vacuum and resuspended in 250 μl bi-distilled water. This desalted peptide mixture was then fractionated in 10 fractions by isoelectrofocusing using a Microrotofor device (Biorad), on an in-solution pH gradient from pH 3 to pH 10. The resulting fractions were then desalted on Stage Tip and the eluates dried and reconstituted to 50 μl of 0.5% acetic acid in water.

Each fraction was analyzed in duplicate on a LC-MS/MS system (Eksigent nanoLC [Eksigent] and LTQ-Orbitrap Velos [Thermo]), using a 155 minutes gradient ranging from 5% to 60% of solvent B (80% acetonitrile, 0.1% formic acid; solvent A = 5% acetonitrile, 0.1% formic acid). Samples for the salt stress analysis were analyzed on the same LC-MS/MS system, but with a gradient lasting 255 minutes.

Resulting raw data were analyzed using the MaxQuant proteomics pipeline v1.1.36 [24] and an in-house database containing swissprot protein data was used. Carbamidomethylation of cysteins and oxidation of methionins were chosen as static and variable modifications respectively; mass tolerance was set to 7 ppm for the precursor and 0.5 Da for the fragment masses; FDR was estimated by the frequency of hits from a decoy database, which was established by inverting the dataset used for proteome analysis and swapping each arginine and lysine with the preceding amino acid [42].

### Metabolomic sample preparation and analysis

For metabolite analysis 2x5ml were filtered through PVDF filter membranes (0.6 μm) (Millipore) using a membrane pump with 10 mbar vacuum pressure. Filters with cells were frozen immediately in liquid nitrogen and stored at −80 °C. The frozen filters were extracted with 2 ml of prechilled extraction buffer (MeOH:CHCl3:H2O; 5:2:1) containing ^13^ C-Sorbitol 2 ug/ml (Sigma) as internal standard under rotation at 4 °C overnight. The solution was centrifuged to spin down insoluble parts. 500 μl of the supernatant were dried under vacuum and stored at −20 °C for metabolomic analysis. Samples were prepared for GC-MS analysis as described previously [19]. The GC-MS chromatograms were processed with the Chromatof software (LECO). Data matrices for relative quantification were extracted from the mass spectra using MetMax software [21]. For statistical analysis intensities were corrected by internal standard and cell-number. Data processing was performed with Microsoft Excel and R, PCA analysis was performed with TigrMev.

For ^13^ C-incorporation study possible reaction products originating from arginine were identified using KEGG and ChlamyCyc [4] (See Figure 4). If the substance was found in our samples the mass shift between no label and full label was identified for multiple fragments per substance using the GMD (http://gmd.mpimp-golm.mpg.de/). Following mass isotopomers were extracted to calculate the ^13^ C label incorporation rate: Putrescine (4TMS) [86/87, 100/101/174/175], Proline (2TMS) [142/146], Glutamic acid (3TMS) [84/85, 100/101, 156/157, 246/248], Ornithine (3TMS)[174,175]. The tool MetMax extracted the intensities for all masses within the given ranges from the exported mass spectra. The percentage of label is calculated by dividing the intensity of the heavy mass by the intensity of the light mass and subtracting the natural ^13^ C abundance calculated from the control.

## Misc

Guido Mastrobuoni, Susann Irgang and Matthias Pietzke contributed equally to the work

## Authors' contributions

GM, performed proteomics analyses and data analysis; SI, performed the time course experiment; MP, analyzed the metabolites; HA, calculated protein half-lives and corrected the manuscript; MW, performed data analysis and calculations; WS, made substantial contributions to conception and design; SK, planned the experiment, analyzed the data, wrote the manuscript. All authors read and approved the final manuscript.

## Supplementary Material

Additional file 5 Further information is present in additional material, which contains supplemental Figures 1, 2 and 3 including legends. Click here for file

Additional file 1 **Table 1**contains all information about the identified proteins in the three salt conditions. Click here for file

Additional file 2 **Table 2**contains the data from reproducibility test for abundance index calculation. Click here for file

Additional file 3 **Table 3**contains all the proteins identified from the 8 h and 24 h samples in the 0 mM condition, after fractionation, including their H/L ratios. Click here for file

Additional file 4 **Table 3**contains the protein half-lives for the protein at 3, 8 and 24 hours.All the raw files form the MS analysis are available for download in mzML format at ftp://bbc.mdc-berlin.de/ gmastrob. Click here for file
