# Supplementary material for: Proteome dynamics and early salt stress response of the photosynthetic organism Chlamydomonas reinhardtii
Source: BMC Genomics. 2012 May 31;13:215. doi: 10.1186/1471-2164-13-215 (PMC3444938; doi:10.1186/1471-2164-13-215)
Supplement: Additional file 5 — Further information is present in additional material, which contains supplemental Figures 1, 2 and 3 including legends. [file 1471-2164-13-215-S5.doc]

**Additional material**

**Additional figure 1,** Rate of ^13^C arginine incorporation in proteins of WT (CC-125) or arg- (CC-1618) *Chlamydomonas* after 2 respectively 12 hours. The graph shows the percentage of ^13^C arginine incorporation of the identified peptides.


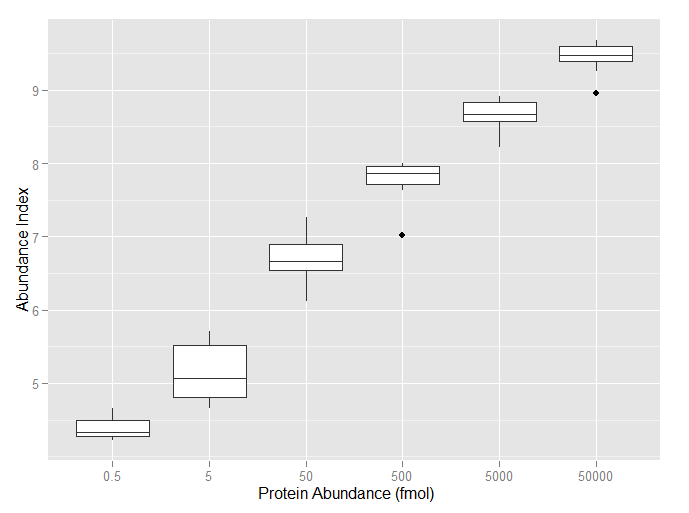


**Additional figure 2**, Abundance index of proteins of the human protein standard 2 measured by shot gun proteomics.

**Additional figure 3,** Relative abundance of proteins from the arginine to proline interconversion pathway at start time (0 mM), 1h (100 mM and 150 mM) and 24h (100 mM and 150 mM).
